# Women’s experience of the quality of care in institutional delivery: evidence from a prospective study in rural south India

**DOI:** 10.1186/1753-6561-6-S1-P3

**Published:** 2012-01-16

**Authors:** Asha Kilaru, Baneen Karachiwala, Zoe Matthews

**Affiliations:** 1 Belaku Trust, Bangalore, India

## Introduction

In the context of rising institutional births in India the question often raised is the quality of care in increasingly crowded labour wards. The success of a policy that encourages hospital birth depends on improved standards of medical care, and also crucially, an adequate level of service satisfaction on the part of women and their families. Not only does quality of care influence health outcomes, it is also likely to affect the understanding, utilisation, perceptions of interpersonal aspects of care and the content of services. These were the aims of our study.

## Methods

A prospective study (n = 642) was undertaken from 2007 to 2009 in 80 villages in a Ramanagara *taluk* (an administrative sub-division of a district) of Karnataka situated in a range of 50 km to 80 km from the state capital of Bangalore. 39 villages were selected in a stratified random sample according to the distance to the primary health centres. An additional 41 villages adjacent to the randomly selected villages were purposively chosen to meet the enrolment target.

## Results

Findings show that 80% of the women delivered in a range of institutions. *Taluk* (*administrative sub-division of the district*) hospitals conducted twice the number of deliveries compared to primary health centres (PHC) and health sub centres. Auxiliary Nurse Midwives (ANM) and nurses handled about 90% of the PHC deliveries, with doctors playing a primary role at the *taluk* and tertiary care hospitals. About 18% of women in the sample delivered at home and the majority with unskilled birth attendants.

A large percentage of families (43%) changed their mind about where the delivery should take place, mostly after labour began. Referrals account for only 25% of those who changed, and other reasons include fear of complications or expecting that the health centre would be closed.

Women's perceptions of provider's interpersonal communication, respect for privacy and confidentiality, and comfort in asking questions show predictable as well unexpected differences between public and private services.

About 35% of institutional deliveries were augmented with oxytocin, and 62% of women left health centres in 6 hours of less, and most without postpartum or newborn care advice. Stratification by caste show that women from scheduled caste or tribe (SC or ST) are experiencing poorer quality of care.

## Discussion

In light of the current policy that aims to achieve 100% institutional deliveries, our data show that high percentages of women receiving poor quality of care and that differences in public *versus* private care are not uniformly in favour of the latter. This may discourage future institutional contacts and limit the intended impact on maternal and newborn mortality and morbidity.

Furthermore, in a state such as Karnataka that performs relatively better than the national average on most health indicators, equity analyses need to explore disparities beyond mortality rates to understand quality of care experienced by women from poor and low caste families.

